# Local disease–ecosystem–livelihood dynamics: reflections from comparative case studies in Africa

**DOI:** 10.1098/rstb.2016.0163

**Published:** 2017-06-05

**Authors:** Melissa Leach, Bernard Bett, M. Said, Salome Bukachi, Rosemary Sang, Neil Anderson, Noreen Machila, Joanna Kuleszo, Kathryn Schaten, Vupenyu Dzingirai, Lindiwe Mangwanya, Yaa Ntiamoa-Baidu, Elaine Lawson, Kofi Amponsah-Mensah, Lina M. Moses, Annie Wilkinson, Donald S. Grant, James Koninga

**Affiliations:** 1 Institute for Development Studies, University of Sussex, Brighton BN1 9RE, UK; 2 International Livestock Research Institute, Nairobi, Kenya; 3 University of Nairobi, Nairobi, Kenya; 4 Kenya Medical Research Institute, Nairobi, Kenya; 5 Royal (Dick) School of Veterinary Studies and the Roslin Institute, University of Edinburgh, Edinburgh, UK; 6 College of Medicine and Veterinary Medicine, University of Edinburgh, Edinburgh, UK; 7 Geography and Environment, University of Southampton, Southampton, UK; 8 University of Edinburgh, Edinburgh, UK; 9 Applied Social Sciences, University of Zimbabwe, Harare, Zimbabwe; 10 University of Zimbabwe, Harare, Zimbabwe; 11 University of Ghana, Legon, Greater Accra, Ghana; 12 Department of Microbiology and Immunology, Tulane University, New Orleans, LA, USA; 13 Kenema Government Hospital, Kenema, Sierra Leone

**Keywords:** zoonosis, ecosystem, livelihoods, disease, Africa

## Abstract

This article explores the implications for human health of local interactions between disease, ecosystems and livelihoods. Five interdisciplinary case studies addressed zoonotic diseases in African settings: Rift Valley fever (RVF) in Kenya, human African trypanosomiasis in Zambia and Zimbabwe, Lassa fever in Sierra Leone and henipaviruses in Ghana. Each explored how ecological changes and human–ecosystem interactions affect pathogen dynamics and hence the likelihood of zoonotic spillover and transmission, and how socially differentiated peoples’ interactions with ecosystems and animals affect their exposure to disease. Cross-case analysis highlights how these dynamics vary by ecosystem type, across a range from humid forest to semi-arid savannah; the significance of interacting temporal and spatial scales; and the importance of mosaic and patch dynamics. Ecosystem interactions and services central to different people's livelihoods and well-being include pastoralism and agro-pastoralism, commercial and subsistence crop farming, hunting, collecting food, fuelwood and medicines, and cultural practices. There are synergies, but also tensions and trade-offs, between ecosystem changes that benefit livelihoods and affect disease. Understanding these can inform ‘One Health’ approaches towards managing ecosystems in ways that reduce disease risks and burdens.

This article is part of the themed issue ‘One Health for a changing world: zoonoses, ecosystems and human well-being’.

## 1. Introduction

Health is a critical aspect of human well-being, interacting with material and social relations to contribute to people's freedoms and choices. Globally, the interaction between human health and the health of the environment is increasingly recognized, along with acknowledgement that healthy ecosystems and healthy people go together [1]. ‘One Health’ discourse and practice integrates animal health into the equation. In this context, zoonotic diseases, emerging or re-emerging as public health problems at the people–wildlife–livestock interface, have become a major focus of scientific and policy attention. While much concern is driven by their capacity to result in global disease outbreaks, from pandemic influenzas to Ebola and Zika virus epidemics, there is growing attention to (once) neglected tropical diseases. In many parts of Africa, for instance, evidence is accumulating of the major impacts of zoonotic diseases, especially on people who are already poor [2].

Much research examines zoonotic disease emergence and impacts at a global scale, tracking and modelling large-scale relationships between ecosystem change, populations and animal and vector habitats, and the relationships with disease outbreaks [3]. There has been relatively less attention to the detailed local interactions between people, disease, animals and ecosystems, untangling the complex dynamics of local systems. Yet it is these local disease–ecosystem–livelihood dynamics that underlie and add up to wider patterns of change, as they interact with larger-scale drivers whether in environment, economy or demography [4]. Evidence and understanding of local system interactions is also a critical basis for informing scenarios and decision-making processes around policy, practice or institutional change geared to improving policy and practice, designing and implementing ‘One Health’ interventions that work in real-world settings.

Research by the Dynamic Drivers of Disease in Africa Consortium during 2012–2016 sought to fill this critical gap. A series of case studies focused on local system contexts and interactions in relation to particular zoonotic diseases in African settings. Each asked: how do ecological changes (e.g. in biodiversity, vegetation and habitat, water) and human–ecosystem interactions affect pathogen dynamics and hence the likelihood of zoonotic spillover and transmission? How do different peoples’ interactions with ecosystems and animals, in the context of their daily lives, livelihoods and socio-economic activities, affect their exposure to disease? How do social differences—by gender, age, wealth, occupation—affect these interactions? What are the synergies, but also tensions and trade-offs, between ecosystem interactions that are important for livelihoods, and those that put people at risk of disease?

**Figure 1.**
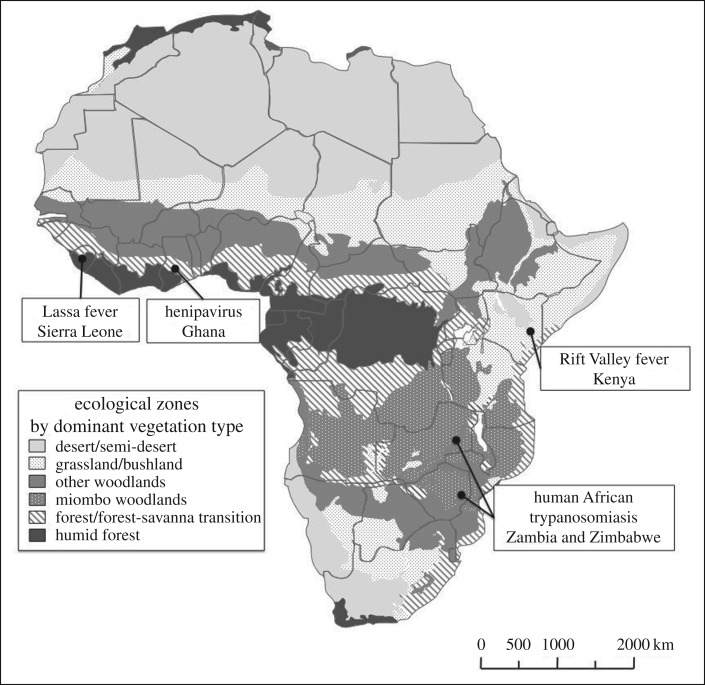
Case study sites in contrasting African ecosystems.

We investigated these interactions through case studies focusing on four diseases in five local systems: Rift Valley fever (RVF) in Kenya, human African trypanosomiasis in Zambia and Zimbabwe, Lassa fever in Sierra Leone and henipaviruses in Ghana. These cases were chosen because, in common, the diseases all require persistent animal reservoir host(s) and zoonotic spillover to cause human disease (unlike viruses of longer zoonotic origin such as HIV/AIDS); yet they involve contrasting transmission routes, ecosystem types and environmental–livelihood dynamics, facilitating a fuller and more comparative understanding. The modes of animal–human transmission considered cover those that are direct (from bats in the case of henipavirus, rodents in the case of Lassa fever and domestic livestock in the case of RVF) and indirect, via insect vectors (mosquitoes in the case of RVF and tsetse flies in the case of trypanosomiasis). The cases also involve a range of non-human vertebrate hosts (both wild and domestic) and degrees of reliance on them. Case study sites are located across ecological zones associated with rainfall gradients and dominant vegetation types, from semi-arid savannah in Kenya, through wooded miombo savannah in Zambia and Zimbabwe and forest–savannah transition in Sierra Leone, to humid forest in Ghana (figure 1). The cases therefore enable a comparative exploration of a range of disease–ecosystem dynamics. The case studies also represent different local contexts of people–livelihood–ecosystem interaction, from a rural and urban contrast in Ghana for the henipavirus case; a village–garden–farm landscape in Sierra Leone for the Lassa case; a contrast between a dry pastoral rangeland and irrigated agriculture in Kenya for the RVF case; and an ecotone between plateau and valley, correlated with changing agricultural and wildlife dynamics in Zambia and Zimbabwe for the trypanosomiasis case.

Each of these case studies was investigated by an interdisciplinary team bringing together medical, veterinary, environmental and social scientists. Methods included ecological and animal population surveys; pathogen/antibody sampling in animal and human populations, with laboratory analysis; socio-economic and livelihood surveys; narrative interviews and focus group discussions (FGDs); ethnographic observations; participatory mapping, ranking and scoring exercises, and the use of secondary data sources—including published literature, government and health centre records, satellite data and spatially referenced databases. Methodological applications, combinations and sequences were adapted to the specificities of each case, as described briefly below. Space constraints in a multi-case paper mean that not all methodological details are able to be presented here, but are cross-referenced to other publications. Notably and in common, however, each case study team combined and triangulated among methods to gain a multi-dimensional picture of disease–ecosystem–livelihood dynamics, including as perceived and experienced by different local people themselves.

The article first presents the setting, specific problem focus and key findings of each of the case studies. It then looks across them to draw out cross-cutting insights around several themes: the relationship between disease and ecosystem dynamics across space and time; interactions between differentiated livelihoods, ecosystems and disease risk; and synergies and trade-offs in using and managing ecosystems for livelihoods and disease. The analysis in turn suggests a number of implications for policy and practice.

## 2. Local disease–ecosystem–livelihood dynamics: five case studies

### (a) Rift Valley fever in Kenya

Rift Valley fever (RVF) is a disease of sheep, goats, cattle and camels caused by a virus carried by *Aedes* mosquitoes. It can also be transmitted to people through the body fluids of infected animals. Outbreaks occur episodically every 5–15 years following periods of above-normal precipitation, often associated with the *El Niño*/Southern Oscillation (ENSO) weather phenomenon [5]. The virus (RVFv) is thought to be maintained during the inter-epidemic periods in eggs of infected *Aedes* mosquitoes, which can survive for several years in dry soil [6] but require heavy rainfall and/or floods to emerge. Ecosystem change, particularly the introduction of flood irrigation and dams in areas that already harbour RVFv, is thus likely to provide conditions for RVFv occurrence and endemicity.

In livestock, RVF causes abortions, stillbirths and the death of young animals, and so severely affects livestock productivity and herd viability, and hence pastoral livelihoods. In people, it causes a flu-like illness which can on occasion be severe or fatal. Much attention has focused on large epidemic occurrences of RVF. In contrast, low-level endemic transmission of RVF is known to be common in rural areas of semi-arid Kenya. The case study investigated how ecosystem changes linked especially to the expansion of irrigation were affecting RVF transmission, and the impacts on people and animals in the context of their interactions with these ecosystems.

**Figure 2.**
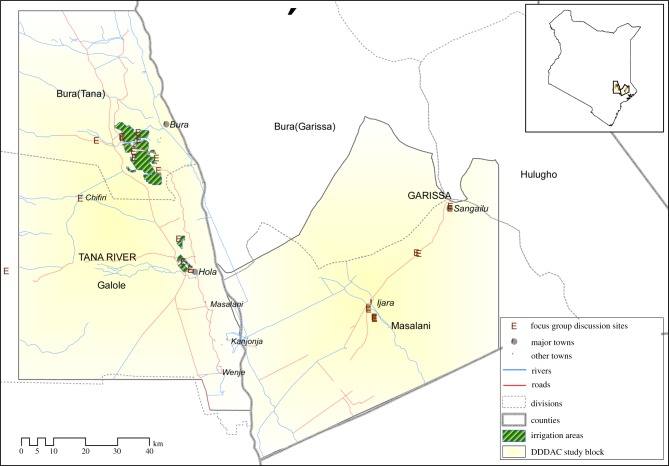
RVF case study site in north-east Kenya.

The study focused on riverine and irrigated areas (Bura and Hola, Tana River County) and pastoral areas (Ijara and Sangailu, Garissa County) in north-east Kenya (figure 2), among people from multiple ethnic communities including the Pokomo, Orma and Somali. It compared land cover and land-use changes, types of mosquitoes present and their densities, peoples’ livelihoods, knowledge and disease control practices, and seroprevalence of the virus in people and livestock in irrigated and pastoral areas.

The study found a major increase in the area under irrigation in the Tana River site compared to Ijara, the area that was being used for pastoral production. Analysis of land-use and land-cover changes between 1975, just before one of the irrigation schemes (Bura) was developed, and 2010 showed a shift from a landscape of mainly open trees, open shrubs, herbaceous vegetation on flooded land and bushlands. By 2010, a number of key habitats had been lost including closed trees (100%), open to closed herbaceous vegetation (100%), bushlands (−36%) and open trees (−30%). A large increase in land cover was in cropland/irrigation where there was an increase of more than 1,400%, followed by open trees on temporally flooded land (50%) and herbaceous vegetation on flooded land (48%).

The study also demonstrated an increase in mosquito abundance in the irrigated areas. Mosquito sampling was carried out in four repeated cross-sectional surveys in irrigated and non-irrigated areas that took a period of 1 year to cover all the seasons. Mosquito larvae were also collected from all the open water bodies within the irrigation scheme and transported to the laboratory where they were reared to adults, identified and screened for viruses. Both primary and secondary vectors of RVFv, including *Aedes mcintoshi*, *Ae. ochraceus*, *Ae. tricholabis* and *Culex pipiens*, were sampled in the irrigated farms. Multivariate models fitted to the data showed that controlling for season and humidity, the irrigated farms had significantly higher densities of mosquitoes than the pastoral areas (table 1). The study also demonstrated that drainage canals in the irrigated area supported the breeding of many mosquito species. When these mosquitoes were screened for arboviruses using standard molecular characterization techniques, eight Ndumu viruses were identified. These viruses also cause febrile illnesses in humans.

**Table 1.** Outputs of a geostatistical model illustrating the effects of land use, season and humidity on mosquito population densities. The regression parameters are mean and percentile ranges (2.5–97.5%) of posterior distributions of fixed and random effects (Deviance Information Citerion estimates for models with and without spatial effect: 702.50 verses 726.96; LULC, land use/land cover).

variable	levels	mean	percentile range	
			2.5%	97.5%
site/LULC	farm—riverine area	−0.16	−1.08	0.77
	village—riverine area	−0.45	−1.25	0.34
	village—irrigation scheme	−0.86	−1.19	−0.53
	pastoral rangeland	−2.27	−2.99	−1.55
	irrigated farm	0.00		
season	very wet	1.84	1.23	2.46
	wet	0.20	−0.17	0.57
	dry	0.00		
humidity		0.03	0.03	0.04
model hyperparameters:				
precision for the Gaussian		1.23	1.01	1.48
Theta1		−6.30	−8.89	−3.99
Theta2		4.42	3.06	5.92

Focus group discussions were held to identify and characterize wealth categories and determine livelihood practices that predisposed people to mosquito-borne infections. A total of 42 FGDs involving 411 people (194 women and 217 men) were conducted: 14 in irrigated sites, 12 in agro-pastoral sites and 16 in pastoral sites. Wealth categories were described based on criteria and thresholds defined by the participants themselves. These included: types of livestock kept, education level achieved, type of housing used, household items owned, source of energy, livelihood sources, and access to food, water and health services. Overall, the median percentages and the 10th and 90th percentile of households that were classified under poor, moderate and rich categories were 56% (15.0–88%), 29.5% (10–61) and 12.5% (2–30%), respectively. This trend was consistent across sites. Notably, however, irrigated areas had a much higher proportion of households in the ‘poor’ category compared to the other sites (74% compared with 38.5% in agro-pastoral and 56.5% in pastoral areas). This could reflect the importance of livestock ownership as a local signifier of wealth, and its absence in the irrigated areas—as well as the high proportion of people in the irrigated areas working as relatively low-paid or casual labourers.

Complementing the focus group discussions, participatory mapping was used to determine ways in which people in pastoral and irrigated areas interacted with their ecosystems as they pursued their livelihood activities. Households in the pastoral sites engaged in livestock husbandry and sale of fodder, trees, firewood and water. In the agro-pastoral sites, crop farming, livestock husbandry and charcoal burning, in that order, were identified as the key livelihood activities while in irrigated areas, livelihood activities entailed crop farming, paid employment (formal/casual), charcoal burning and the sale of firewood and water. These activities varied by wealth; in all three sites, wealthier households either had formal employment or ran profitable businesses, while those in the middle- and low-wealth categories worked as labourers in irrigated farms, herded animals for pay or fetched water, firewood, grass and building materials including poles for sale. Ecosystem interactions and livelihood activities also varied by gender. In irrigated agriculture, women were more engaged in planting and weeding, while men were mainly involved in watering and spraying of the crops against pests. In agro-pastoral and pastoral sites, there was a clear separation of the various livestock activities implemented by women, men, young boys and girls.

These ecosystem interactions in turn brought differentiated vulnerability to RVF. A sero-epidemiological survey showed that the risk of exposure to RVFv was higher in people in irrigated areas compared to pastoral and riverine areas. This is illustrated in table 2, although the difference was not statistically significant.

**Table 2.** Association between land use and seroprevalence of RVFv in people. (Outputs of a geostatistical model. The regression parameters are mean and percentile ranges (2.5–97.5%) of posterior distributions of fixed and random effects.)

variable	posterior		
		percentile range	
	mean	2.5%	97.5%
fixed effects—land use			
irrigation scheme	0.29	−0.34	0.94
riverine area	0.12	−0.70	0.92
pastoral area	0.00		
random effect—SPDE2 Model			
Theta1 for i	−2.12	−3.12	−1.09
Theta2 for i	0.68	−0.40	1.69

Thus irrigation, as a major form of ecosystem and land-use change in semi-arid Kenya, has brought increases in wealth for a few. However, these benefits have not been widely shared and the majority of women and men remain engaged in livelihood activities that keep them in poverty. At the same time, irrigation has contributed to reductions in well-being by increasing vulnerability to RVFv infection.

### (b) Trypanosomiasis in Zambia

**Figure 3.**
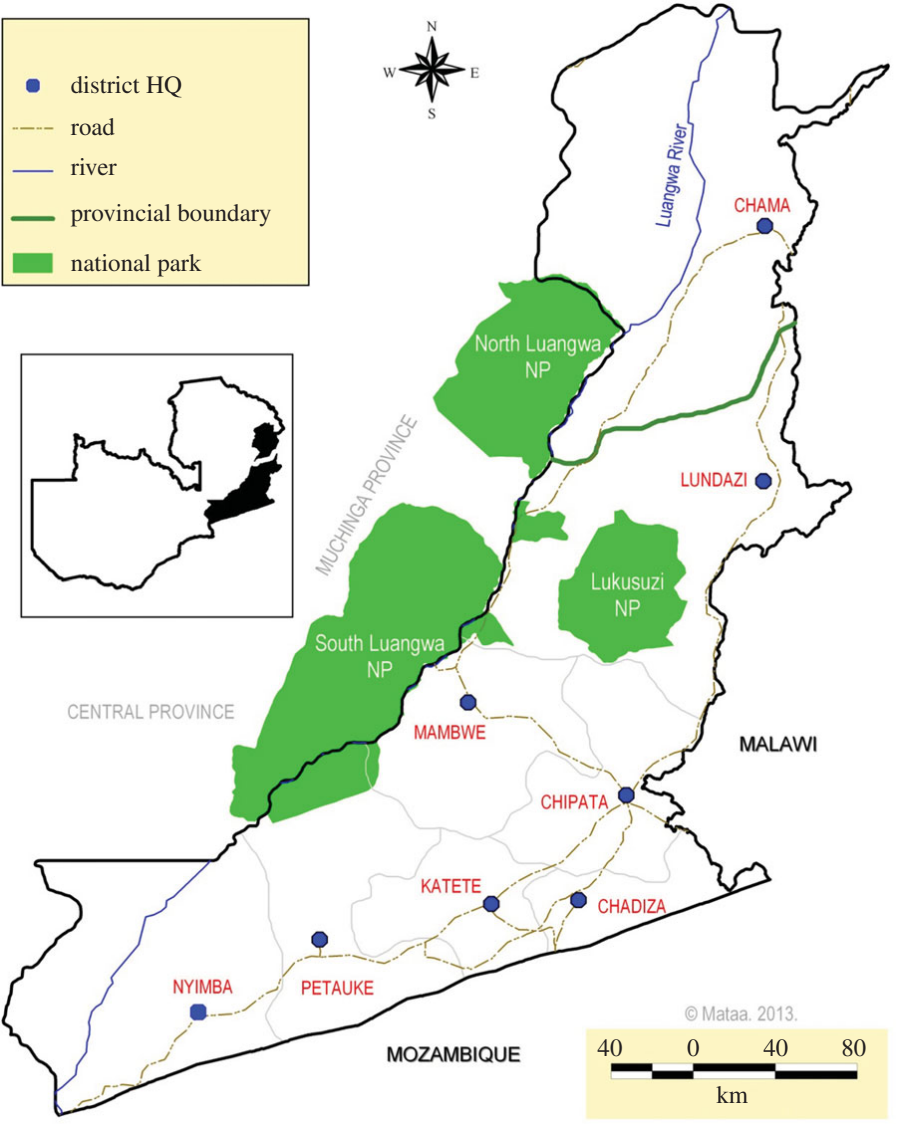
Trypanosomiasis study site in the Luangwa Valley, Zambia.

Human African trypanosomiasis (HAT) is a zoonotic disease caused by the protozoan parasite *Trypanosoma brucei rhodesiense*, transmitted by the tsetse fly (*Glossina* species). The Luangwa Valley in Eastern Zambia (figure 3) is a well-recognized focus for disease outbreaks which occur sporadically as a spillover from a widespread reservoir in both domestic and wild animals [7,8]. It is an area of high biodiversity with four national parks which are bounded by game management areas (GMAs) in which regulated utilization of natural resources (primarily through professional safari hunting) is permitted. Human population densities in these GMAs are relatively low. Tsetse-transmitted trypanosomes also cause disease in animal populations, with several pathogenic species recognized as causing African animal trypanosomiasis (AAT) in domesticated animals. There has been an almost complete absence of livestock keeping due to the high trypanosomiasis challenge, and livelihood and cultural practices focus on wildlife utilization. Agricultural activities are permitted, but land and resource use systems have remained relatively consistent over the last century.

Over the last few decades there has been an influx of people into the mid-Luangwa Valley around the tourist centre of Mfuwe and south towards Katete (figure 3) on the eastern plateau bounding the valley [9]. New settlements have been created and livestock have been introduced in large numbers. Cotton production has become widespread along with other forms of cultivation with resultant changes in land cover. As the distribution of tsetse species is largely determined by climatic and environmental factors such as temperature, vegetation cover and availability of vertebrate hosts, these changes could have a profound impact on tsetse populations and trypanosomiasis transmission. Similarly, changes in human demographics and behaviour could result in many more people becoming at risk of infection with HAT. Areas such as this where livestock have recently been introduced and where they exist at the interface with wildlife and tsetse populations have been identified as being at particular risk of epidemics [10]. This case study investigated the effect of these ecological and social changes on the epidemiology of HAT, and on the livelihoods and well-being of local communities.

The study area consisted of a transect approximately 75 km long between Mambwe and Katete. Field research included a census in 2012 of all human and domestic animal inhabitants residing along this transect and the surrounding area. A cross-sectional survey to estimate the prevalence of trypanosomiasis (HAT and AAT) in humans, cattle, sheep, goats, pigs and dogs was conducted in 2013 using molecular methods for diagnosis [11,12], and data were compared to a similar study in 2005 [10]. A detailed human livelihoods and well-being survey in 211 households, as well as a smaller human movements survey, were completed [13]. Participatory methods, including 28 key informant interviews, nine focus group discussions and 19 participatory mapping sessions and transect walks, were used to assess local community knowledge and practices. Tsetse surveys were conducted in June and November 2013 using standard tsetse sampling techniques for the region (Epsilon traps and black screen fly rounds). Remotely sensed satellite imagery was used to characterize and quantify land-cover change in the study area from 1990 to 2013. Geostatistical modelling is being used to investigate how environmental covariates influence the prevalence of trypanosomiasis in cattle and the density of *Glossina morsitans* tsetse populations.

The census identified 3717 households within the study area, supporting 17 656 people, showing an increase in the population, although more modest than expected. Ethnic groups represented included Kunda, Chewa, Ngoni, Nsenga, Bemba, Tumbuka and Bisa people. Local migration is common with 62% of households reporting that they had moved location, including 37% within Mambwe District. In-migration was less common than expected, with 17% migrating from other parts of Eastern Province and 8% from other parts of Zambia or neighbouring countries. The main driver for in-migration has been poor soil fertility on the eastern plateau and pressure on land due to the high human population density.

Livelihood practices have also changed. Livestock are now kept in large numbers with 14 914 domestic animals in the study area including 3169 cattle. Agricultural activities have become increasingly important. Cotton is the main cash crop, grown by 85% of households; maize and groundnuts are the main food crops. Land cover was found to have changed significantly, largely due to the clearing of new areas for cultivation. Within a 5 km zone around the households studied, the proportion of agricultural land has increased from 10% to a third in the past 25 years. The main source of energy is wood; harvesting it also contributes to the loss of woodland (table 3).

**Table 3.** Change in the area of agricultural land in the Zambian study site.

year	study area		Mambwe District	
	land area under agriculture (ha)	percentage of total area under agriculture	land area under agriculture (ha)	percentage of total area under agriculture
1990	10 000	12	20 000	3
2000	14 000	16	26 000	4
2013	26 000	30	55 000	10

**Figure 4.**
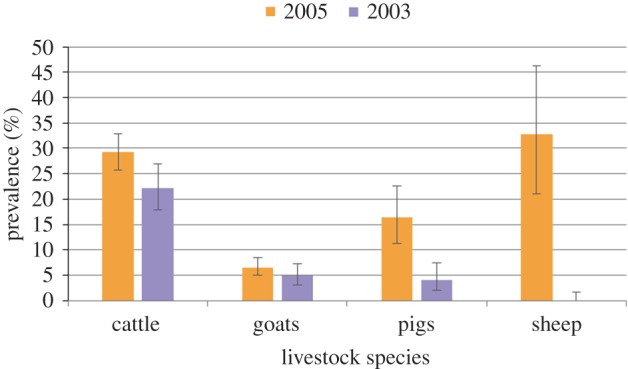
Estimated prevalence of all pathogenic trypanosome species detected in 2013 compared with 2005 (this includes both HAT and AAT). Error bars display 95% confidence intervals.

**Figure 5.**
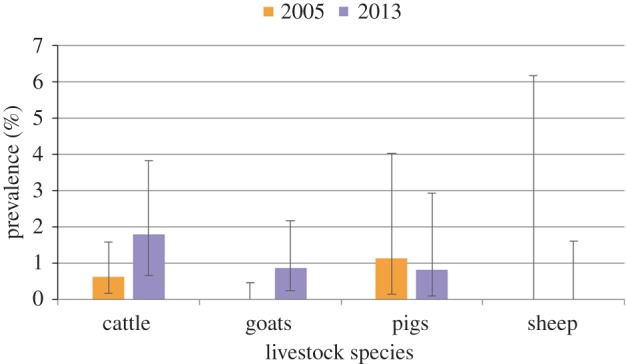
Estimated prevalence of *T. brucei* s.l. in 2013 compared with 2005 (*T. brucei* s.l. includes the human-infective subspecies *T. b. rhodesiense*). Error bars display 95% confidence intervals.

The overall prevalence of trypanosomiasis in livestock (HAT and AAT) declined from 16.99% (95% CI: 15.23–18.86) in 2005 to 8.44% (95% CI: 6.98–10.10) in 2013 (figure 4). In contrast, the prevalence of *T. brucei* s.l. (which includes the human infective *T. b. rhodesiense* sub-species) increased from 0.35% (95% CI: 0.13–0.77) in 2005 to 0.94% (95% CI: 0.49–1.63) in 2013 (figure 5). Survey data are not available for other years, so it is possible that these changes may reflect annual variation in prevalence, rather than a long-term trend. No human-infective *T. b. rhodesiense* was identified in the human or animal populations sampled, but test sensitivity is a diagnostic constraint and occasional cases are reported locally. The apparent density of tsetse detected was relatively low and the majority of flies sampled were *G. morsitans morsitans* with very few *G. pallidipes*, most probably reflecting the relative resilience of *G. m. morsitans* to ecosystem modification.

As land cover and livelihoods have changed towards agriculture, it is probable that this has contributed to the relatively low apparent density of tsetse and a reduction in the combined prevalence of all trypanosome species in livestock. However, this reduction in prevalence was due to a reduction in the species causing AAT rather than *T. brucei* s.l. Due to difficulties inherent in the diagnosis of the human-infective *T. b. rhodesiense* subspecies in animals, *T. brucei* s.l. is often used to investigate potential HAT infection. Therefore, the ecosystem changes appear to have been beneficial in terms of AAT, but have not reduced the risk of HAT transmission. Given that the number of livestock (and therefore the size of the potential livestock reservoir for HAT) has also increased, the risk to human health could potentially increase in the future despite the reduction in the overall trypanosomiasis prevalence.

Migration patterns are complex with people often moving over short distances. Those who in-migrate and settle at the edge of intact vegetation are more likely to be exposed to the risk of infection through contact with tsetse. In particular, tsetse were primarily detected in the northwest of the study area towards the South Luangwa National Park and Lower Lupande GMA, which still largely contains woodland savannah vegetation; the growing numbers of people living in this interface area, or entering to gather fuelwood and other resources, may be at a heightened risk of contracting HAT, when compared with people living in the centre of settled areas. These important human movement patterns have been captured in an agent-based model currently under development, which predicts two human infections over a six-month simulation period. This is in keeping with the sporadic nature of the disease in the Luangwa Valley.

Thus changing livelihood and ecosystem dynamics linked to population growth, in-migration and a shift from wildlife-based to agricultural activities have been associated with a decline in overall trypanosomiasis risk in animal populations, but the risk of zoonotic trypanosomiasis persists. Those who inhabit and visit the interface areas with woodland and wildlife—interfaces whose location is shifting as land-cover change proceeds—are particularly vulnerable. For in-migrants and others dependent on these zones, positive benefits for livelihoods and well-being from making use of woodland resources also bring the corresponding risk of disease. If tsetse persist in sufficient numbers within the interface zone, people and livestock will continue to be at risk of infection.

### (c) Trypanosomiasis in Zimbabwe

A case study of trypanosomiasis was also conducted in Zimbabwe, revealing a slightly different set of disease–ecosystem–livelihood dynamics in a broadly similar ecological setting. Until the 1990s, the Zambezi Valley—the area between Zambia and Zimbabwe—hosted high tsetse fly populations and experienced frequent trypanosomiasis outbreaks. These were the focus of major, widespread quasi-military control campaigns, initially focused on eradicating wildlife hosts [14], later on eradicating the fly using chemical sprays, baits and traps, and the Sterile Insect Technique (SIT), and more recently drugs and vaccines [15]. The same time period has also seen major changes in vegetation and land cover linked to human population growth, in-migration and settlement, and the expansion of agriculture, especially cash-cropping. It seems likely that these ecosystem–livelihood changes have—perhaps more than official tsetse control efforts—brought about a reduction in tsetse populations and trypanosomiasis cases, in a similar dynamic to that experienced in the Luangwa Valley in Zambia. Nevertheless, the problem persists. Eleven cases of HAT were reported in 2010, three in 2013 and 2014, and one in 2015. It is likely that numbers in reality are much higher since villagers rarely report sleeping sickness, cases reported to local clinics are often missed [16] and some choose to manage the disease traditionally instead [17]. The Zimbabwe case study sought to understand the ecosystem–livelihood dynamics accounting for this persistence of trypanosomiasis risk, and who is vulnerable to it.

**Figure 6.**
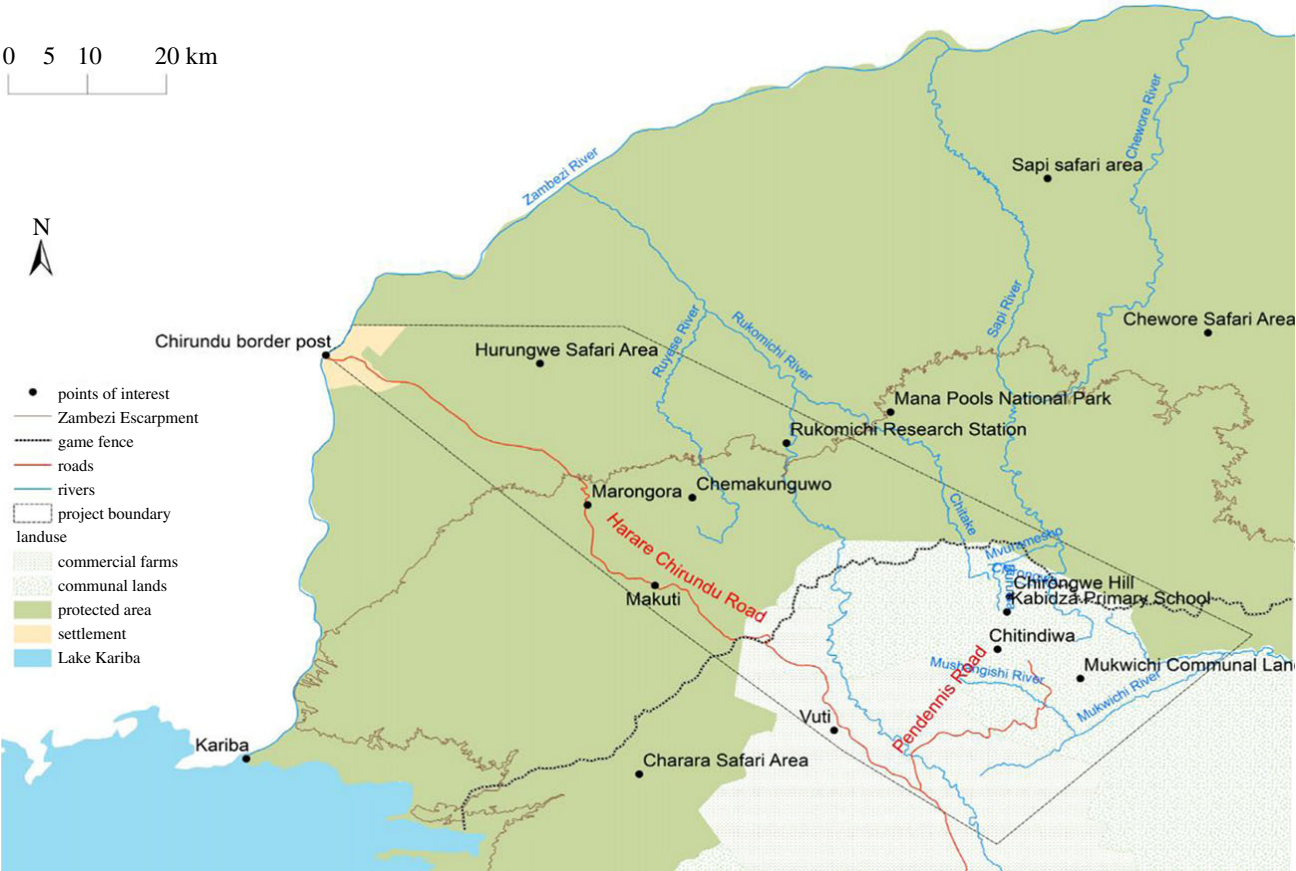
Trypanosomiasis study site in Hurungwe District, Zimbabwe.

The study focused on Hurungwe District (figure 6), characterized by highly biodiverse wooded miombo savannah. A large part of the site is classified as protected areas established in the 1960s including Mana Pools (in the north), Chewore and Sapi Safari areas, in the northeast, and Hurungwe Safari area in the northwest, while others are classified as communal areas and commercial farms. Methods combined Geographic Information System mapping and use of secondary data to map landscape changes with entomological surveys and trapping to map the distribution of tsetse flies. Interviews and participant observation were used to explore socially differentiated interactions with ecosystems and how this related to livelihood and cultural practices. Local knowledge derived from participatory methods was also used to guide the positioning of tsetse fly traps, in order to follow up inhabitants’ own hypotheses about fly prevalence.

The District has undergone fundamental changes in population and land cover over the last few decades. In the 1980s the population was less than 20 000. Settlement was discouraged by the prevalence of trypanosomiasis, and later by protracted guerrilla warfare [18,19]. Since the 1980s, there has been significant in-migration, resulting in a differentiated mix of people pursuing different livelihood activities. First, there are the Korekore people, who still engage in foraging and hunting, with some moving into agriculture. Second, there are long-term migrants, attracted to Hurungwe by land and firewood for commodity production [20]. These migrants dominate the production of tobacco and cotton, threatening Korekore land control [21]. Third, there are ‘squatters’, a disparate group seeing Hurungwe as a refuge—whether fleeing the state, displaced from commercial crop farming areas or retrenched from mining towns following the collapse of the economy. Whatever their origin, squatters are very poor and vulnerable.

**Figure 7.**
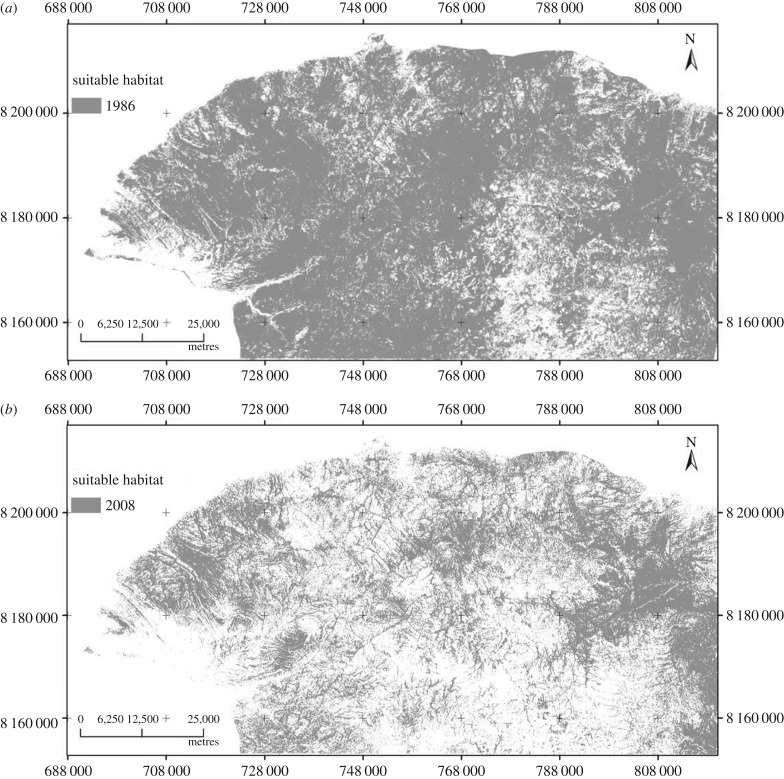
Suitable tsetse fly habitat (suitable habitat, cells with a probability of occurrence of tsetse fly of 0.5 and above; unsuitable habitat, cells with a probability of less than 0.5 [22]) in Hurungwe District, 1986 (*a*) and 2008 (*b*).

While human settlement, in-migration and the introduction of cotton and tobacco farming have generally transformed land cover, there nevertheless remain many patches of woodland, locally termed *tumasango*. These include steep valleys that cannot be accessed for settlement or development. They also include river banks, sacred hills and wetlands where territorial spirits are considered to dwell. In contrast to the cleared homesteads and farmlands, these patches carry wooded vegetation, providing ideal habitats for the tsetse fly. Figure 7 shows these changes in the suitable habitat for tsetse, from widespread woodland cover in 1986, to its concentration in patches in 2008.

**Figure 8.**
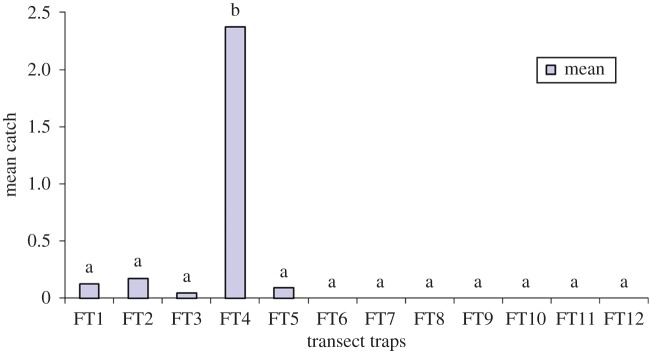
Tsetse fly distribution below and above the escarpment, Hurungwe District. (Online version in colour.)

Entomological surveys confirmed that these patches are not just ideal habitats, but actually contain tsetse flies. Twelve [11] fly traps were deployed over seven [6] months (February to August) in a transect in the Zambezi Valley. The results are shown in figure 8, which suggests a gradient, with tsetse found in transect traps on the wooded valley floor (FT1–3), peaking at the highly wooded escarpment (FT4) and dropping to zero in the settled areas above it (FT6–12). FT4 gave the highest standard deviation, indicating that there was a huge variation in the monthly trap catches in FT4 compared to the other traps.

Epidemiological surveys found a corresponding patchy pattern to disease prevalence. Trypanosomiasis infections were found primarily in livestock inhabiting such patch areas. Where infections were reported in settled areas, they were the result of animal movements. Finally, participatory analysis revealed the same picture. When asked to indicate places where tsetse flies were found, villagers pointed to the Mushagizhe Valley and Chewore River, where as one put it:

These have more tsetse than any other area. Chitindiva (settled area) there is no fly at all, and all you have are mosquitoes. As far as we know, these valleys and river banks are maternity areas of tsetse. The flies are found there anytime of the year (Interview, 2014).

**Figure 9.**
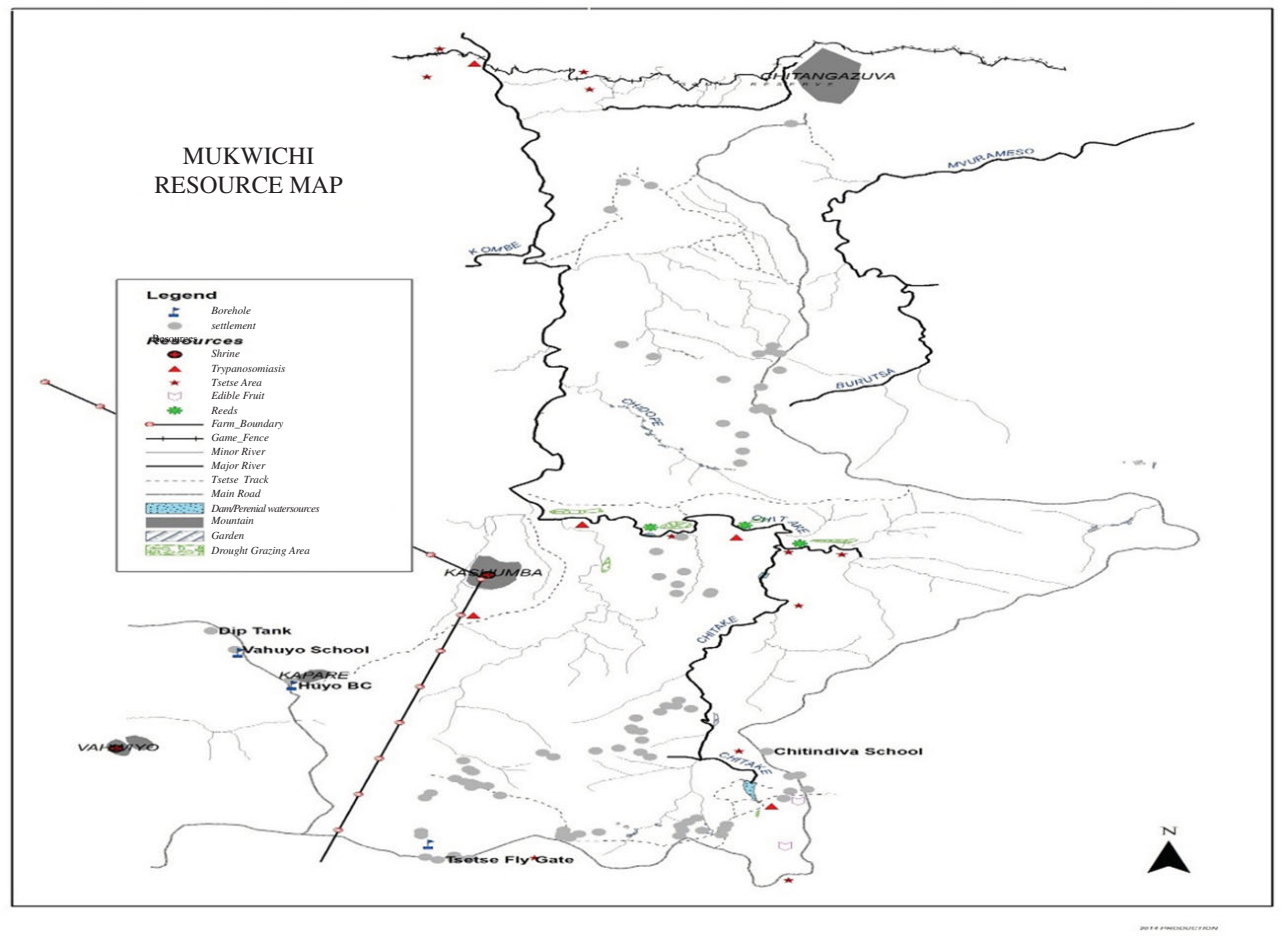
Resources in the Mukwichi area.

Notably, these woodland patches are also rich in terms of resources. As figure 9 illustrates, based on a participatory map of Mukwichi in the communal area, there is always water, due to the perennial rivers and streams in the valleys. There are opportunities for both browsing, grazing and water for livestock, especially valuable between September and early December, the dry season. In some patches there is also high wildlife presence, encouraged by a controversial community conservation project [17]. This brings opportunities for subsistence hunting and, in the dry season, for sport hunting of big game such as elephants and buffaloes. Woodland patches also present opportunities for foraging (fruits, tubers, insects), as well as sites for cultural practices such as pilgrimages to these as sacred places where spirit mediums, central in land fertility and dispute settlement, dwell.

As people seek to access these resources in the context of their livelihoods, they are vulnerable to tsetse fly exposure and HAT risk. The most vulnerable groups include cattle herders, women who gather forest products, hunters and pilgrims. Also vulnerable are recent migrants because they are the main groups to access woodland interfaces, where they seek land for tobacco farming, as well as squatters who sometimes seek to live deep in woodlands where the state authorities cannot reach them.

Thus tsetse and trypanosomiasis persist in Hurungwe, in spite of a history of massive control attempts and a general transformation of land cover away from suitable tsetse habitats. Key are the woodland patches found within a wider dynamic ecosystem, which provide continued habitats for the tsetse fly and a source of exposure to disease for those people who access these patches in their livelihoods.

### (d) Lassa fever in Sierra Leone

Lassa fever (LF) is a haemorrhagic human disease caused by Lassa virus. The disease has a broad spectrum of severity ranging from fever and sore throat, to haemorrhaging, organ failure and death. Humans are incidental hosts and the virus is maintained by transmission and asymptomatic infection of the Natal multimammate rat, *Mastomys natalensis* [23–25]. This rodent is commonly found in agricultural communities throughout sub-Saharan Africa and is responsible for significant crop loss and transmission of a number of pathogens including those that cause plague, leishmaniasis and leptospirosis [26–28]. Despite the rodent's pervasive distribution in Africa, LF appears to be endemic only in West Africa. Recent studies by the Dynamic Drivers of Disease in Africa Consortium (DDDAC) associate the virus with a genetically distinct subgroup, confined by geographical barriers [29].

Within West Africa, LF has a patchy distribution indicative of epizootic cycles in agricultural communities. While human-to-human transmission can occur particularly in clinical settings, another DDDAC study estimated that the majority of LF cases are acquired from rodents [30]. The current case study aimed to assess the impact of land-use variation on small mammal abundance, livelihoods and hence increased exposure to *Mastomys* species.

Centuries of settlement in the region have created a mosaic landscape of villages or towns, surrounded by backyard gardens and anthropogenic forest islands, beyond which lie upland rice fields and fallows, swamps used for rice and vegetables, and forest regrowth. Previous studies have shown that the rodent is most abundant in villages and surrounding backyard gardens, rather than in more distant farmland or forest [31,32]. Few studies have identified an age- or gender-associated risk for LF [33–36], yet research has not been fine grained and suffers from poor incidence and prevalence data. Exposure to the virus is believed to occur through contact with rodent urine/faecal contamination of food, water and surfaces. Most speculation about Lassa exposure has centred on rodent–human interactions in and around homes, while the only recognized rodent-associated risk factor for LF is hunting of rodents for food consumption [37]. However, in Sierra Leone, reported LF incidence fluctuates seasonally, with peaks in the dry season (February–March) and a smaller peak in the rainy season (June/July) [36], suggesting that climatological factors and agricultural labour patterns may impact rodent–human contact. Anthropological literature from the region suggests that exposure in homes and agricultural activity could be differentiated by gendered and age-specific labour and livelihood activities [38], and that villagers prefer to consume ‘bush’ rather than ‘town’ rodents [39]. This raises the likelihood of differentiated but widespread exposure to LF shaped by livelihood-related ecosystem interactions beyond the village.

The case study examined *Mastomys* abundance and human activities in different agricultural land-use types over seasonal time points tied into major agricultural activities. It took place in eastern Sierra Leone in the districts of Kenema and Kailahun, dominated by Mende-speaking people, where there is a high level of LF incidence. Four communities of varying size were selected, each having a history of LF activity, with subsistence farming as the primary livelihood activity for most residents. We originally planned to collect ecological and social science data in all communities at eight time points over 2 years, but activities were disrupted from June 2014 to July 2015 due to the Ebola epidemic. As a result, collections were reduced to four separate time points, each coinciding with key agricultural activities (table 4).

**Table 4.** Seasonal agricultural activity cycles for Lassa fever case study time points.

time point	activities	
	upland mixed crop cycle	swamp rice cycle
Nov 2013	harvest	harvest
Mar 2014	soil prep—clearing and burning land	vegetable gardening
May 2014	soil prep, planting	minimal activity
Aug 2015	weeding	weeding

Small mammal trapping was carried out in all land-use areas as identified by agricultural/environmental researchers and information from villagers (table 5). A standard number of live-capture traps were set in each area (excluding villages and nearby backyard gardens) for three nights and the GPS location of each trap recorded. All small mammals captured were identified to genus level, marked with an ear tag displaying a unique identifier and released. In subsequent data collection periods, traps were set in approximately the same location, even if the land-use type changed (i.e. cultivated field to fallow land). Only our final data collection period (August, 2015) included village and backyard garden sampling. In this period, all collected animals were euthanized after sample collection.

**Table 5.** Land-use areas in Lassa fever case study.

land-use category	description
rice swamp	low-lying area with seasonal flooding for rice crops
upland mixed farm	sloped area with good drainage used for growing a variety of crops including rice, maize, groundnuts, cassava, okra and sorghum
young fallow	formerly upland mixed farm left unattended for 1–4 years
old fallow	formerly upland mixed farm left unattended for 5–10 years
cleared land	formerly fallow area that has been cleared of vegetation and possibly burned in preparation for planting
tree crop	heavily shaded cultivated area for cacao and coffee crops
palm plantation	shaded area for palm trees of varying height; crops include palm oil and palm wine
small holder mining	secluded, forested area with extreme land perturbance due to upturning soil, pit digging and panning for diamonds
forest	primary, uncultivated area, often used as cemetery and left mostly unperturbed
backyard garden	area within 10–15 metres of a house where vegetables such as peppers, spring onions, yams and cocoyams, groundnuts and tomatoes are grown
village	clearly delineated with few trees, houses mostly constructed of earth and sticks, metal or thatched roofs

**Figure 10.**
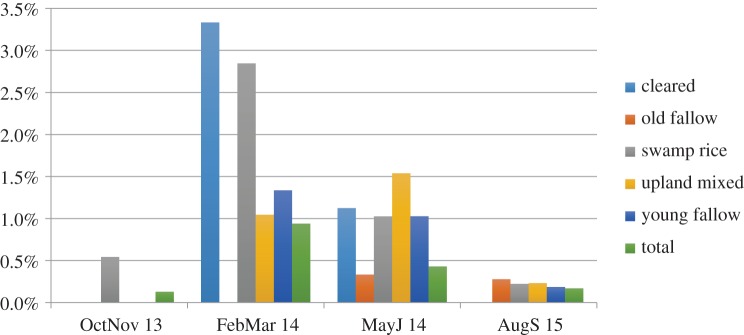
*Mastomys* trap success (number of rodents captured) by agricultural use and time point. Note: no *Mastomys* rodents were captured in tree crop, mining or forest areas.

*Mastomys* rodents were most abundant in villages and backyard gardens when compared to surrounding cultivation and forested areas. In land-use types with multiple samplings (all areas except villages and backyard gardens), no *Mastomys* were found in forests, tree crop or mining areas. The rodents were most abundant during the dry season (February/March 2014), which coincides with historical peaks in LF incidence in this area (figure 10, also [36]). Land-use types with larger numbers of *Mastomys* include recently cleared land and swamp rice areas. This is at a time of extreme human activity-related perturbation of the soil, with implications for transmission. In cleared land, trees are felled by men and the land is cleared by men and boys with machetes and then burned, probably displacing rodents that have burrowed into the soil in these fields. The swamp rice fields are dry during this time and are the site of intensive hands-on work. The soil is ploughed by hand into mounds, by men, sometimes as paid labourers, where additional vegetable gardens are cultivated by women.

*Mastomys* abundance was lowest at the peak of the rainy season (August 2015) across all land-use types. This time period is known locally as the ‘hungry season’, where crops are maturing and little food is available. It is likely there are significant population crashes for rodents during this time. During the harvest, the rodent was found only in rice swamps, where grains are being harvested, and rodents are probably feasting on dropped grains.

The division of labour during these peak times is quite gender- and age-specific. Adult men traditionally form collective work parties to fell trees and clear fallow bush, while women and children gather and bundle wood for cooking fires or sale. They are also responsible for feeding the men's work parties and usually prepare food in the fields. Women are also preparing the vegetable mounds in dried rice swamps. This is a critical livelihood activity for many women, who can sell their vegetables at markets for personal profit.

Certain livelihood activities therefore increase the risk of contact between *Mastomys* rodents and humans and therefore the potential for gendered and age-specific routes of transmission. Notably, these peak times for contact in the agricultural cycle coincide with what have been identified as peaks of LF incidence in this region [35]. There are, moreover, potentially important links—and trade-offs—between gendered livelihood activities and vulnerability to LF which require further investigation.

Vulnerability to LF also relates to rodent consumption, which is in turn shaped by local perceptions and consumption practices. Villagers most commonly identified the ‘long-nosed’ rat, which is found in the town and is said to have a repugnant odour, as the spreader of LF; however, this is a misconception. Based on villagers’ identification of photos, this animal is most probably a shrew belonging to the *Crocidura* genus, and not associated with Lassa virus infection or transmission. It seems that local perceptions of rodents pertain more to where they are observed than to species [38]. Although some people avoid eating ‘town’ rodents or the ‘long-nosed’ rat, such restrictions do not apply to bush rodents, which include *Mastomys.*

### (e) Henipavirus in Ghana

In many rural and urban towns in Ghana, large roosts of bats are found near human habitation. Bats provide important ecosystem services such as seed dispersal, pollination and suppression of arthropod species that would otherwise become pests [40,41]. Bats are also an important source of protein in many parts of Africa, and have been shown to constitute a significant element in the bushmeat commodity chain in Ghana [42–44]. However, bats are known to be reservoirs of several zoonotic pathogens and have been implicated in the transmission of deadly filoviruses such as Ebola and Marburg, lyssaviruses (rabies-like viruses), coronaviruses (e.g. SARS) and paramyxoviruses such as Hendra and Nipah viruses [45–51]. Henipaviruses have been isolated from bats in Australia and Asia, and evidence of infection has been reported in bats from Africa [48,52–55]. Henipaviruses cause encephalitic disease in humans and domestic animals with extremely high case fatality rates [56,57].

Evidence of henipaviruses in human populations has not been established as yet in Ghana; however, there is evidence of henipavirus circulation in bats within the country [48], suggesting the potential for a disease spillover from bats to humans, although no formal risk assessments have yet been carried out. The case study sought a better understanding of possible points of disease risk by exploring the prevalence and location of bats in Ghana, how people interact with bats in the context of their livelihoods and use of ecosystems, how this differed by social group and between rural and urban areas, and people's perceptions of bats and disease.

**Figure 11.**
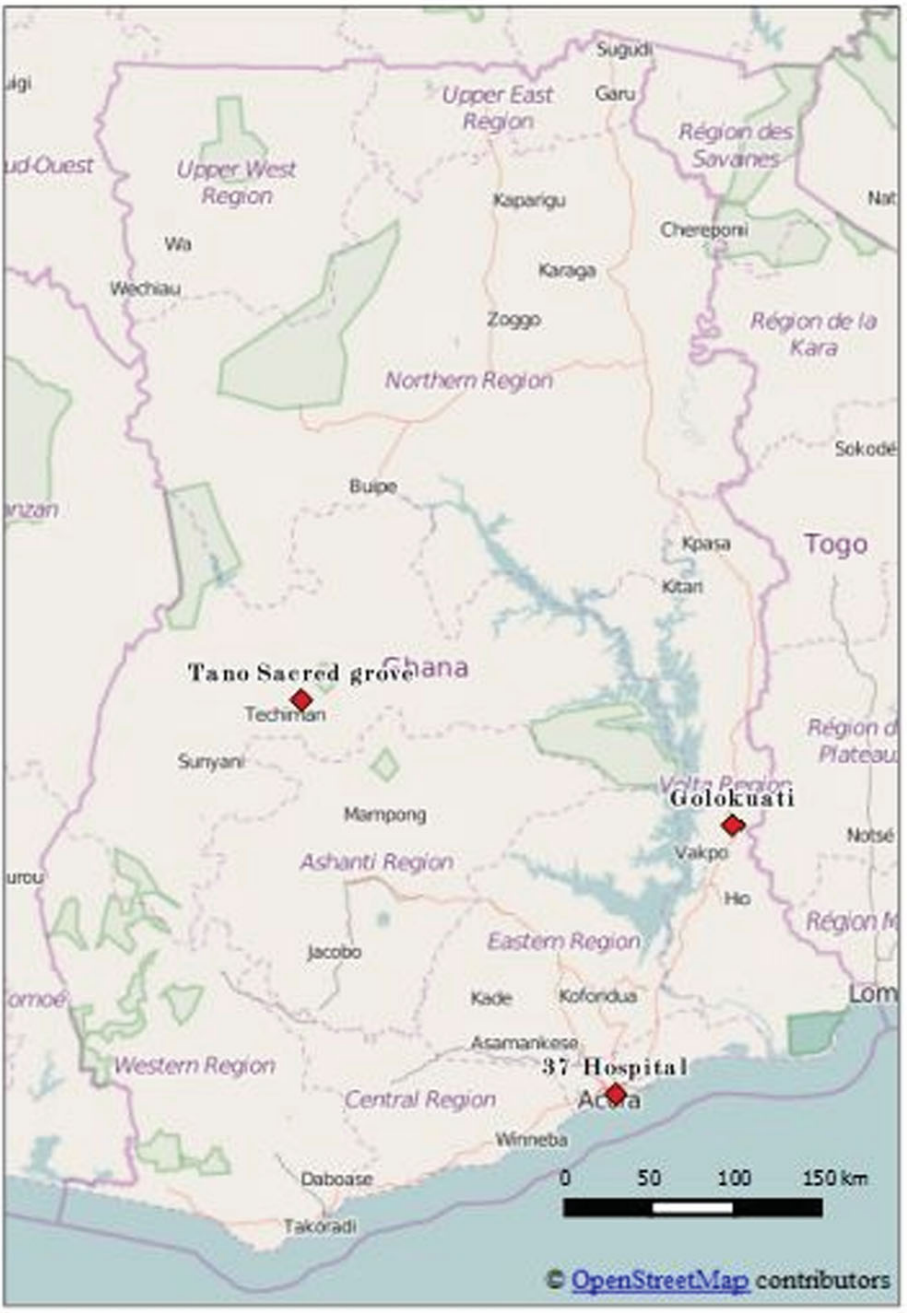
Henipavirus study sites in Ghana.

The focal study sites comprised two rural (Golokuati and Tanoboase) and one urban (37 Military Hospital, Accra) localities (figure 11), described in detail by Lawson *et al*. [58]. Their common characteristic was the presence of large roosts of fruit bats. The 37 Military Hospital is situated in the centre of Accra near a transport terminal, and people living and working there are from a mixture of ethnic groups. Large numbers of fruit bats roost on mahogany trees along the main road in front of the hospital and within the hospital compound, thus exposing patients, hospital visitors and the general public using the transport terminal to bat urine and faeces. Bat roosts could also be found on trees in residential areas and on the grounds of the Parks and Gardens department located near the hospital. The Tano sacred grove is located in Tanoboase, a small farming community along the Techiman–Kintampo road in the Brong-Ahafo Region, dominated by people speaking the Bono version of Akan, but also with migrants from the north of the country speaking other languages. Sacred groves are patches of forest set aside by local communities and protected by traditional norms for a variety of religious and sociocultural purposes [58]. The site is estimated to support over 2 million bats during the peak season. The inhabitants are mainly small-holder farmers cultivating food and cash crops such as yam, maize, plantain and cashew. Ve-Golokuati, whose people are primarily Ewe, is located along the Tema–Jasikan Road, within the forest–savannah transition zone. A large population of bats roosts in mango (*Mangifera sp*), fig (*Ficus* sp) and neem (*Azadirachta indica*) trees in the town, within school and church compounds, market places and people's homes.

For the ecological studies, primary data were collected through direct field observations and a nation-wide survey using citizen science approaches and literature to map fruit-bat distribution. Ecological data on bat species were collected through mist-netting and radio-tracking. Focus group discussions, participatory landscape mapping, transect walks and semi-structured interviews (a total of 340; 164 women and 176 men) were used to document livelihood practices, human–bat interactions, and people's perceptions of bats and disease (see [59] for details). The study also involved surveillance of bats, domestic animals and human populations for evidence of henipavirus seroprevalence.

**Figure 12.**
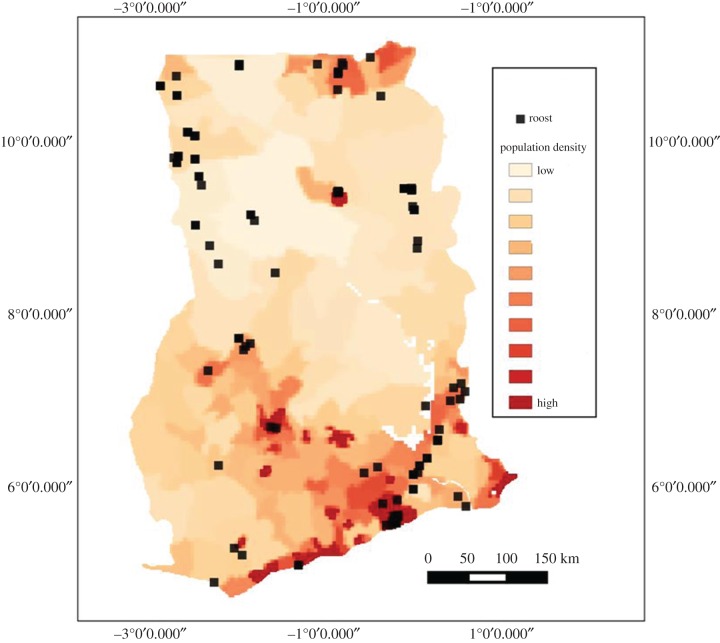
Location of fruit-bat roosts in Ghana in relation to population distribution. Population base map from the Center for International Earth Science Information Network [60].

Thirteen species of fruit bats have been recorded in Ghana; they occur across the country in all ecological zones. Nearly 6000 individual fruit bats, belonging to ten species, were captured in mist-nets. The two species *Eidolon helvum* and *Epomophorus gambianus* were the most abundant, accounting for over 75% of captures. Other common bat species encountered were *Micropteropus pusillus, Rousettus aegyptiacus* and *Epomops franqueti*. Bat roosts were reported from 86 locations all over the country, commonly in densely populated areas (figure 12). Of the total roosts investigated, 95% occurred within 50 m of buildings/homes and farmlands. Several of the large bat roosts occurred in cities and towns.

Livelihood activities centred around farming in the rural sites, while in all sites people were variously involved in petty trading, artisanal/construction work, food processing/trading as well as government work (teaching/health service/military service). In their livelihoods and everyday life and work, people interacted with bats in diverse ways, directly and indirectly. Direct exposure to the possibility of disease spillover involved bat hunting, which was common, particularly at Tano and the 37 Military Hospital area, as well as processing fresh bat meat for consumption, selling fresh bat meat and consuming undercooked bat meat.

Indirect exposure to disease risks resulted from regular exposure to bats, bat faecal droppings, bat urine and bat saliva through livelihood activities such as farming and fruit collection (e.g. handling of fruits half-eaten by bats), domestic animal husbandry, where domestic animals such as pigs are housed under bat roosts and often feed on fruits half-eaten by bats, and trading and selling of wares under bat roosts—a common occurrence at the 37 Military Hospital and the Golokuati township where the town market was held under a huge bat roost. Indirect exposure to bats also occurred through social activities including recreation and community meetings under trees on which bats roost. In the Golokuati township, people actually lived with bats in their homes. Bats roosted on trees in people's courtyards and people went about their daily household chores, such as food preparation, washing and social activities (e.g. women gathering to plait their hair), under bat roosts. The system of rainwater harvesting in open containers for domestic use and drinking at Golokuati also posed potential risk, as the water could be contaminated easily with bat faeces.

Four particularly high-risk groups with potential exposure to henipavirus emerged, linked to where and how they interact with rural and urban ecosystems and bats. These are, first, fruit farmers (especially cashew farmers); more farmers than others hunted bats and also handled fresh bat meat. Second are hunters; third, traders; and fourth, people who live or work close to bat roosts (such as residents living with bats in Ve-Golokuati and health professionals and hospital maintenance staff working at the 37 Military Hospital).

There is evidence that bat hunting occurred at all three sites, mainly as a secondary occupation, but only a small number (5%) of respondents actually indicated that they hunted bats. Bat hunting was primarily a male activity and the association between gender and bat hunting was highly significant (*χ*^2^ test, *p* < 0.002). Men were also more likely to butcher and handle fresh bat meat. Approximately 40% of respondents consumed bat meat and there was a significant association between gender and bat meat consumption, with a higher proportion of men eating bat meat than women (*χ*^2^ test, *p* < 0.05). However, the study showed that other factors such as the size of the bats as well as the level of protection by the local authorities also played a role in bat hunting and consumption. For example, *E. helvum* is more highly preferred by hunters because of its bigger size, while bats roosting at the 37 Military Hospital were more strongly protected, as hunting within the hospital premises was strictly prohibited unless specifically authorized and organized by the soldiers.

Interestingly, people's perception of disease risk associated with bats was very low (table 6). As many as 62% of respondents perceived no risk or ‘small risk’ from hunting, butchering, cooking and eating poorly prepared bat meat. Indeed a number of people associated eating of bat meat with a range of health benefits.

**Table 6.** Perception of degree of risk associated with bat-related activities.

activity	perceived degree of risk posed			
	none or small risk		significant/serious risk	
	frequency	percentage	frequency	percentage
butchering/preparing	83	15.26	59	10.85
eating poorly prepared meat	81	14.89	55	10.11
hunting	82	15.07	71	13.05
cooking	91	16.73	22	4.04
total	337	61.95	207	38.05

Thus close proximity of bat roosts to human dwellings and intense human–bat interactions linked to people's livelihood activities and social and cultural interactions with ecosystems present multiple opportunities for disease spillover from bats to humans—even if this is not currently widely understood by local residents.

The study has established widespread distribution of *E. helvum* and other fruit bats in the country (figure 12), and the team has found evidence of high seroprevalence of henipavirus in *E. helvum* colonies [61]; therefore it seems reasonable to postulate that zoonotic spillover of henipavirus occurs. The absence of detected disease outbreaks in these communities so far may be the result of challenges in diagnostic surveillance, as well as of unknown or variable pathogenicity of African henipaviruses for humans. The ongoing analysis of human blood samples should enable further interrogation of this proposition.

## 3. Comparative and cross-cutting insights

The case studies reveal a variety of ways in which disease–ecosystem–livelihood dynamics are unfolding in local systems, with implications for the risks of zoonotic spillover to different groups of people.

First, ecosystem dynamics and land-cover change affect the prevalence of animal reservoirs, vectors and their habitats, influencing the possibilities for disease transmission. These dynamics involve diverse, interacting temporal scales. Thus, in the case of trypanosomiasis in both Zambia and Zimbabwe, a timescale of several decades has seen the transformation of wooded miombo savannah to farmed landscapes with reduced tsetse fly prevalence. A similar timescale in semi-arid Kenya has seen a major expansion of irrigated land, increasing the prevalence of RVF-transmitting mosquitoes. Demographic changes (increasing populations and in-migration) have been important drivers of land use change in all these cases, as have been the expansion of commercial farming and cash-cropping.

In contrast, the Sierra Leone and Ghana cases show more overall ecosystem continuity, in mosaics of forest, savannah, fallow and settlement land that have characterized Upper Guinean ecosystems for decades, if not centuries [62]. These present a relative degree of stability in habitats for disease-reservoir wildlife (rodents and bats), coexisting with settlements and farmed land in long-established anthropogenic landscapes. However, changes within this continuity include the expansion of small-scale horticulture (in Sierra Leone) and tree cropping (in both Ghana and Sierra Leone), and the growth of towns and peri-urban landscapes. In different ways both trends have increased the availability of peri-domestic habitats for disease-carrying rodents and bats.

Such long-term landscape change can be punctuated by shorter-term shocks. Several of the case study diseases show both endemic patterns of continuous spillover, combined with outbreaks, which in turn can be related to sudden or episodic ecosystem changes. Thus RVF outbreaks are associated with episodes of above-normal rainfall linked to *El Niño* cycles. Outbreaks of haemorrhagic fevers (such as Lassa fever, as well as Ebola) have elsewhere been associated with exceptionally sudden-onset dry season conditions, although our study timescales were insufficient to identify such outbreak dynamics in the case studies themselves. Conducted over a 2–3 year timescale, however, the case studies have been able to reveal seasonal changes in the prevalence of animal disease reservoirs and vectors. Both the Zimbabwe and Zambia study sites have one rainy season with quite pronounced seasonal effects on both tsetse and wildlife populations. In Kenya, populations of RVF-carrying mosquito populations vary annually across the seasonal cycle, while in the case of Sierra Leone, rodent habitats shift with seasonal cycles of upland farm–fallow and swamp rice–vegetable garden dynamics. Notably, in these latter cases the key seasonal dynamics with respect to disease depend on seasonally varying agricultural and agro-pastoral land use as well as climate.

Second, spatial dynamics intersect with these temporal ones. All the case studies involved a spatially identified local system, although bounded in different ways in keeping with the problem to be addressed: contrasting urban/rural settlements and their surrounding landscapes in Sierra Leone and Ghana; a pair of districts with contrasting levels of irrigation in Kenya; and contiguous wildlife/pastoral/agricultural areas in Zambia and Zimbabwe. Within each, though, spatial dynamics are a key part of the unfolding story of ecosystem–animal–disease interactions. In Zambia, these relate mainly to land use differentials along a gradient in altitude; in Zimbabwe, to the patch dynamics of woodland amidst agro-pastoral land use; in semi-arid Kenya, to contrasts between irrigated commercially farmed areas, and those dominated by rangelands; and in Ghana and Sierra Leone, to the location and size of settlements, and patches of different types of land use within shifting mosaics, in ecological settings where rodents and bats occupy peri-domestic spaces. Whatever the details, all the cases highlight the importance of micro-differences within local systems, and of mosaic and patch dynamics, as ecological, human and animal population factors interact.

Third, the critical question with respect to human vulnerability to zoonotic spillover concerns how people interact with these dynamic ecosystems, and the extent to which these interactions expose them to pathogen-carrying wildlife, livestock or vectors. The case studies reveal a wide array of ecosystem interactions that are central to people's livelihoods and well-being. Shaped by varied political economies and social relations, and with local variation in the resources available, valued and used, these include pastoralism and agro-pastoralism (Kenya, Zimbabwe, Zambia); commercial and subsistence farming (all cases); hunting (all cases); and the collection of food, firewood and medicines (all cases). As the examples of sacred forests in Ghana and pilgrimage sites in Zimbabwe illustrate, particular sites within ecosystems are also visited as part of cultural and ritual practices. Everyday living and movement in a settlement or landscape, for social and non-directly ecosystem-dependent livelihood purposes such as trade, can also bring people into contact with pathogen-carrying animals—as in the cases of bat roosts in Ghana and Lassa-carrying rodents in Sierra Leonean villages. While revealing the multiplicity and diversity of livelihood-related ecosystem interactions, however, the cases also point to significant social differences in livelihood profiles and ecosystem use, which in turn can suggest particular, socially differentiated vulnerabilities to disease. The examples of women gardeners’ vulnerability to Lassa fever, or the vulnerability of squatters and hunters drawn to tsetse-inhabited woodland patches to trypanosomiasis in Zimbabwe, exemplify the broader point that ‘who gets sick and why’ depends on social and livelihood difference as these intersect with ecologies [63].

A close understanding of the interactions between ecosystem, livelihood and disease dynamics in turn reveals synergies, but also tensions and potential trade-offs, between patterns of system change that are positive in terms of ecosystems, of livelihoods and of disease. For instance in Zambia and Zimbabwe, landscape transformation for commercial farming has been synergistic with a reduction of trypanosomiasis risk. The retention and use of woodland patches is vital for some people's livelihoods—but brings the trade-off of tsetse exposure. In Kenya, irrigation has brought commercial agricultural profits and employment, at least to some people; but it has also enhanced RVF transmission. In Sierra Leone, dry season vegetable gardening is a vital addition to women's livelihoods and economic independence, but also exposes them to Lassa fever. In Ghana, the use of bats for bushmeat is a valuable source of livelihood and well-being for many people—but also brings disease risk. Such synergies and trade-offs can be helpfully clarified in the concepts and language of ecosystem services [64,65]. People interact with and make use of a variety of ecosystem services in the course of their lives and livelihoods, which may be provisioning, regulating, supporting or cultural services. Yet in so doing, they may also experience the ‘ecosystem disservice’ of disease. Interventions that enhance some ecosystem services may also increase the ecosystem disservice of disease risk (as in irrigation which enhances the service of hydrological regulation, but increases the disservice of RVF transmission). Disease regulation can also be conceptualized as an ecosystem service; framed thus, the land-use changes in the RVF case, for instance, can be seen to have reduced the wildlife and dryland vegetation conditions that kept RVF transmission to people and livestock at a relatively low level, and irrigation has disrupted such disease regulation.

## 4. Conclusion and implications

Such tensions and trade-offs are not amenable to simple solutions, precisely because multiple interactions and values are at stake. To take one example, a proposal simply to cull bats in Ghana because they carry disease would rightly be (and indeed has been) met with objections because of the value of bats as a source of vital ecosystem services—from pollination to provision of bushmeat and other livelihood resources. Instead, a detailed understanding of such interactions, synergies, tensions and their implications for different people should be seen as the basis for a more informed approach towards managing ecosystems in sustainable ways that reduce disease risks and burdens.

This could include, first, a more differentiated, targeted approach to interventions. Disease control, surveillance and monitoring need not always take a full system, landscape-level approach, but will often be more effective—and efficient and cost-effective—if focused on the parts of systems where problems exist—such as patches with high tsetse populations, or particular field types linked to Lassa fever exposure. Equally, interventions and monitoring can be time-targeted, focused on the seasons or weather events that pose most risk. Second, an understanding of local ecosystem–animal–livelihood–disease interactions provides both a justification and basis to look beyond single-sector approaches, to locally attuned ‘One Health’ approaches. Thus addressing Lassa fever in Sierra Leone, our analysis suggests, could benefit from joined-up thinking and action between health and agricultural practitioners, in identifying potential solutions that link disease control with the management of rodents as agricultural pests. Likewise, the RVF case study points to the value of improved irrigation technologies and better management of water (e.g. better drainage) to prevent vector-borne zoonoses in addition to the standard RVF interventions.

Finally, approaches need to be informed by local knowledge. Understanding and acting on local disease–ecosystem–livelihood interactions, and taking account of the distribution of risks and impacts across different groups of people, requires collaboration between scientists, policymakers, practitioners and crucially the users of ecosystems themselves. This is a vital basis not just for understanding how these interactions are unfolding with what implications, but also for deliberating on potential solutions, in ways that bring a nuanced appreciation of who will gain or lose.

## Data accessibility

The case studies in this article draw on primary data collected as part of the Dynamic Drivers of Disease in Africa Consortium's country case studies. Appropriate datasets are in the process of being archived in line with guidance issued by the Ecosystem Services and Poverty Alleviation programme. Environmental data (e.g. vegetation and animal surveys) will be available in the NERC Environmental Information Data Centre. Socio-economic data such as transcripts of focus group discussions and socio-economic surveys will be archived in appropriate repositories of the UK Data Service.
